# Identification and functional analysis of novel centrosomal proteins to study their implication in human disease

**DOI:** 10.1186/2046-2530-1-S1-P28

**Published:** 2012-11-16

**Authors:** L Jakobsen, JM Schrøder, K Vanselow, EA Nigg, E Lundberg, J Andersen

**Affiliations:** 1 University of Southern Denmark, Denmark; 2 Biozentrum, University of Basal, Switzerland; 3 School of Biotechnology, AlbaNova University Center, Royal Institute of Technology (KTH), Stockholm, Sweden

## 

The physiological importance of cilia is underscored by the ever-growing list of ‘ciliopathies' associated with ciliary dysfunction. The many overlapping phenotypes of these syndromes might reflect the complexity of the ciliary proteome and the numerous functions of cilia. Likewise, dysfunction of a number of centrosomal proteins has been linked to human diseases. As centrosomes and cilia are closely linked, centrosomal abnormalities can also cause disease by affecting cilia formation, for example by affecting the docking of the basal body to the cell membrane, transport processes relying on microtubule interactions with the centrosome, targeting of membrane vesicles or the intraflagellar transport required for cilia assembly. It might thus be that a number of diseases could be more correctly referred to as ‘centrosomeopathies’. Despite the identification of many centrosomal and ciliary proteins, it is still not known how many of these components interact and function. We have recently characterized the human centrosome proteome in depth using a quantitative mass spectrometry-based method in combination with an antibody-based screen, identifying several novel and candidate centrosomal proteins. The thereby obtained human centrosome proteome provides an excellent basis for further experiments to elucidate the multiple functions of the centrosome, including its role in human disease such as ciliopathies and cancer development. We are currently undertaking more functional experiments based on the improved centrosome proteome to investigate a possible role of these proteins in processes such as centriole duplication and ciliogenesis, both of which are processes with links to human disease.

